# Techniques for investigating lncRNA transcript functions in neurodevelopment

**DOI:** 10.1038/s41380-023-02377-5

**Published:** 2023-12-25

**Authors:** Tara Srinivas, Edilene Siqueira, Sonia Guil

**Affiliations:** 1 Josep Carreras Leukaemia Research Institute (IJC), 08916, Badalona, Barcelona, Catalonia Spain; 2 Germans Trias i Pujol Health Science Research Institute, 08916, Badalona, Barcelona, Catalonia Spain

**Keywords:** Neuroscience, Molecular biology, Biological techniques, Genetics

## Abstract

Long noncoding RNAs (lncRNAs) are sequences of 200 nucleotides or more that are transcribed from a large portion of the mammalian genome. While hypothesized to have a variety of biological roles, many lncRNAs remain largely functionally uncharacterized due to unique challenges associated with their investigation. For example, some lncRNAs overlap with other genomic loci, are expressed in a cell-type-specific manner, and/or are differentially processed at the post-transcriptional level. The mammalian CNS contains a vast diversity of lncRNAs, and lncRNAs are highly abundant in the mammalian brain. However, interrogating lncRNA function in models of the CNS, particularly in vivo, can be complex and challenging. Here we review the breadth of methods used to investigate lncRNAs in the CNS, their merits, and the understanding they can provide with respect to neurodevelopment and pathophysiology. We discuss remaining challenges in the field and provide recommendations to assay lncRNAs based on current methods.

## Introduction

### lncRNAs and neurodevelopment: rationale for the present review

Less than 2% of mammalian genomic DNA is ultimately translated into proteins [1, 2]. By contrast, roughly 70-90% is transcribed to produce a vast population of non-protein coding RNAs (ncRNAs). ncRNAs consist of multiple classes, including ribosomal RNAs (rRNAs, which compose ribosomes), transfer RNAs (tRNAs, which transport amino acids to ribosomal machinery), small nuclear RNAs (snRNAs, which mediate pre-mRNA splicing), and micro RNAs (miRNAs, which silence RNA via complementary sequence pairing). Less understood are the long noncoding RNAs (lncRNAs), which are characterized by a length greater than 200 nucleotides and an absence of functional open reading frames (ORFs). The GENCODE project has identified 17,957 human lncRNA genes, with some additional lncRNAs produced from human pseudogenes (which number 15,000) [3–6]. Over 100,000 human lncRNAs have been recorded [7, 8]. LncRNAs have only recently begun to garner attention as potentially functional molecules in the cell, since they can form secondary and tertiary structures, interact with nucleic acids and proteins, and are conserved at the promoter level. They may act as biological decoys (for miRNAs, for example), chromatin modifiers, scaffolds, and more [9]. Genome-wide association studies have moreover uncovered that >90% of disease- and trait-associated variants lie in noncoding regions [10].

The CNS contains a highly diverse set of lncRNAs [11], and the brain alone expresses roughly 40% of known mammalian lncRNAs [12, 13]. Interestingly, the most rapidly evolving loci of the primate genome contain sequences encoding ncRNAs with roles in neural development [14], and lncRNAs are expressed at high levels in the mammalian brain [15, 16]. Indeed, in human ESCs, neurogenesis and differentiation are blocked by siRNA-mediated knockdown of individual lncRNAs [17]. The nuclear lncRNA *MALAT1*, enriched in post-mitotic neurons, is known to regulate synaptic gene expression [18], and the CNS-enriched lncRNA *Paupar* acts in *trans* along with the epigenetic regulatory protein KAP1 to regulate olfactory bulb neurogenesis [19]. lncRNAs are thus clearly implicated in brain and developmental processes, yet the mechanisms by which they modulate these processes remain unclear. As the pathophysiology of many neurodevelopmental disorders remains uncharacterized, uncovering the contributions of CNS-enriched transcripts like lncRNAs to neurodevelopmental pathogenesis will be extremely useful, not only in the field of neurobiology but also in the clinical sphere. We therefore review techniques and associated challenges for investigating lncRNA functions in mammalian neurodevelopment.

### A brief overview of lncRNA genomic, structural, and biological features

**Fig. 1:**
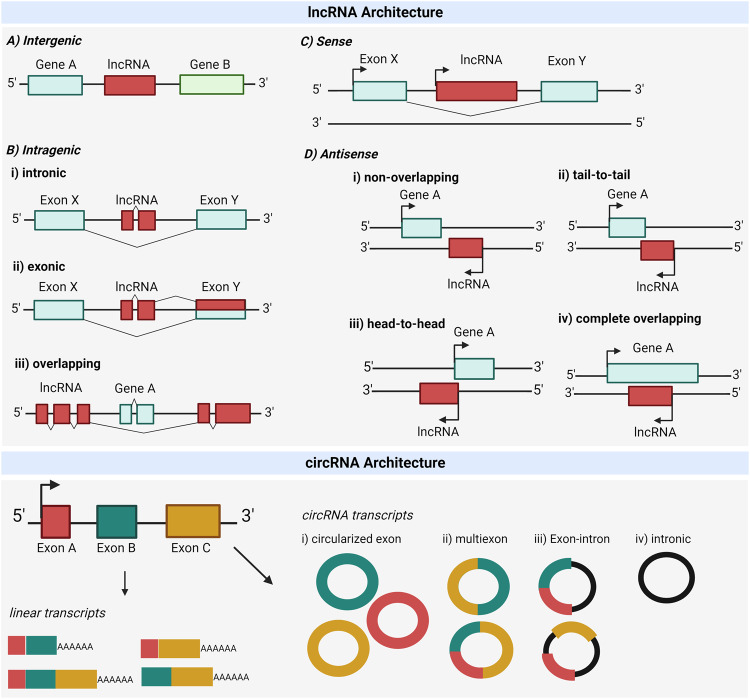
lncRNA and circRNA categories based on their transcription direction and overlapping regions. The gene architecture of lncRNAs refers to their localization in reference to nearby coding genes. Top panel: lncRNA genes can be **A** intragenic, if the lncRNA gene is located between two other genes, or **B** intragenic, when it is located within a gene. In this case, the transcripts are (i) intronic, when located between two exons, (ii) exonic if they overlap with at least one of the exons of the host gene, or (iii) overlapping, when their sequence completely surrounds the exons of a coding gene. In terms of their transcriptional direction, ncRNAs are **C** sense, when transcribed on the same strand as the host gene, or **D** antisense, when transcribed on the opposite strand of a sense strand-derived RNA. Antisense transcripts are further categorized based on their degree of overlap as (i) non*-*overlapping, when the lncRNA gene has no overlapping region with the neighboring gene, (ii) tail-to-tail or convergent transcription when lncRNA transcription overlaps in the 3′UTR with the neighboring gene, (iii) head-to-head or divergent transcription when the overlapping region is in the 5′UTR, and, (iv) complete overlapping, when the lncRNA transcription region overlaps fully with the coding gene. NB: Intragenic lncRNAs can also be classed as sense or antisense, depending on the strand from which they originate. Bottom panel: circRNA architecture is categorized according to (i) circularized exons, when a single exon forms a circularized RNA, (ii) multiexons, when multiple exons circularize to form a circRNA, (iii) exon-intron, when the circRNA is formed by exonic and intronic regions of the neighbouring gene, or (iv) intronic, when it is formed by intronic regions. Created with Biorender.

Several iterations of lncRNA classification based on genomic position [6, 7, 20] have resulted in the knowledge that lncRNAs may be transcribed, processed, and/or derived from such regions throughout the genome as introns, repetitive elements, protein-coding loci, and 3′ UTR sequences. LncRNAs also include intergenic ncRNAs (lincRNAs, which do not overlap with coding genes), and sense and antisense transcripts that may overlap with other genes (Fig. 1). Like mRNAs, lncRNAs are often transcribed by RNA Polymerase II (Pol II), alternatively spliced, capped at their 5′ ends by a 7-methyl guanosine, and/or polyadenylated at their 3′ ends. However, unlike mRNAs, many lncRNAs are retained in the nucleus (likely due to their accumulation on chromatin and weak internal splice signals), contain fewer exons, are expressed in a cell-type specific manner, and are less evolutionarily conserved [21].

Several lncRNAs have a repeatable modular structure [22] and/or derived from transposable elements [23] with multiple domains that are thought to mediate their biological functions, including scaffolding of proteins, RNA, and DNA. In the nucleus, such RNA-mediated interactions allow lncRNAs to participate in transcription and chromatin modification, remodeling, and organization, while in the cytoplasm, they can affect post-translational modifications and the localization and stability of other RNAs or proteins. There is mounting evidence to suggest that lncRNAs can be further defined by distinct structural motifs that may give rise to previously uncharacterized biological functions [24]. Regarding organ-specific lncRNAs functions, we have previously reviewed the roles and therapeutic potential of lncRNAs in the mammalian CNS, in particular outlining evidence of dysregulated *AK081227* and *BDNF-AS* lncRNA expression in the neurodevelopmental disorder Rett Syndrome. Other lncRNAs have been identified in the pathogenesis of Autism Spectrum Disorder as well [25]. By contrast, this review aims to add new knowledge by outlining the challenges and techniques associated with lncRNA functional investigation in the mammalian CNS and particularly in neurodevelopment.

### Traditional methods and challenges associated with lncRNA experimental investigation

Unbiased detection and analysis of lncRNAs has traditionally taken place using tiling microarrays (in which cDNA is hybridized to overlapping oligonucleotides that cover a chromosomal region or the whole genome), serial analysis of gene expression (SAGE, in which cDNA fragments generated from sequences throughout the transcriptome are cleaved by restriction enzymes, concatenated, and sequenced to provide a snapshot of the transcripts isolated), chromatin immunoprecipitation of actively transcribed loci (in which DNA associated with chromatin subcomponents is isolated and sequenced to infer the loci on which the lncRNA acts), and RNA-seq (in which total RNA is reverse transcribed and sequenced) [26]; currently, most lncRNA annotation is based on RNA-seq. Once a lncRNA is identified, it is often useful to alter its expression to ascertain its function. However, because lncRNAs have a relatively complicated architecture, altering lncRNA expression can be difficult.

Tools to diminish RNA expression have traditionally included programmable nucleases, in which the gene locus undergoes direct mutagenesis. Four types of programmable nucleases exist: meganucleases (MegNs), zinc finger nucleases (ZFNs), transcription activator-like effector nucleases (TALENs), and the clustered regularly interspaced short palindromic repeats (CRISPR)/CRISPR associated protein (CRISPR/Cas) system. The CRISPR/Cas system has become popular for use in high-throughput forward genetic screens, transcription interference (CRISPRi), and gene activation (CRISPRa) as well. While many of these systems have been used to manipulate coding regions [27], few have been attempted specifically on lncRNA loci in neural systems. Another option is post-transcriptional approaches such as RNA interference (RNAi, including siRNA, esiRNA, miRNA, and shRNA) and antisense oligonucleotides (ASOs), which are reviewed with respect to neurodevelopmental applications here.

lncRNA loci present a few distinct challenges with respect to targeted gene editing. Firstly, small insertions and/or deletions (indels) may not be sufficient to cause loss of lncRNA function. As a result, and due to their lack of functional ORFs, many lncRNAs are targeted at or upstream of their promoters. However, lncRNAs can be transcribed from a variety of potentially problematic promoters, including bidirectional promoters that regulate other genes and promoters that are situated within the body of another gene. Furthermore, some lncRNA loci are located within the introns of other genes while others are transcribed antisense to and intersecting with neighboring genes. These considerations make targeting lncRNAs with a~20 nucleotide single guide RNA (sgRNA), ZFN, or TALEN while avoiding effects on neighboring gene expression highly difficult. Defining and/or manipulating functional domains within lncRNAs can be similarly experimentally difficult, as their protein-binding modules and/or targeting sequences can vary between stages of cell development. Separate challenges regarding transcript-level targeting exist, including the sometimes unpredictable nature of lncRNA structure and localization. Additional difficulties arise when assessing the applications of these methods to the CNS, particularly in vivo, where penetration of the blood-brain barrier (BBB) and cell-type-specific targeting must be considered. Here we review successful techniques for functional studies of lncRNAs in the CNS with an eye toward neurodevelopment and promising new methods.

## Understanding techniques to modify lncRNA expression in models of the mammalian CNS

### Genome-level manipulation

#### Overview of programmable nucleases

Genome editing, a pillar of functional genomics, allows the study of a gene’s role in any given tissue, which is especially crucial for lncRNAs. Often, the central question in lncRNA studies is whether they are required for proper viability or development like their mRNA counterparts are [28]. Furthermore, as the brain expresses a rich collection of ncRNAs, the manipulation of multiple types of noncoding molecules, especially lcnRNAs, provides valuable information about the phenotypical consequences of their loss during neural development [29].

Before the development of CRISPR/Cas technology, genome editing relied on homologous recombination (HR), an intrinsic mechanism during meiosis that assures genetic variability. HR is based on the exchange of homologous DNA sequences between paired chromosomes. To purposefully achieve HR, an exogenous donor DNA sequence, homologous to the target site, is donated to the cells in anticipation of a possible meiotic cross-over. Although effective, the method is statistically impracticable [30]. To overcome this caveat, site-specific nucleases have been engineered to induce double-strand breaks (DSB) in the DNA, favoring local DNA repair. The repair machinery can perform two types of correction: the error-prone non-homologous end joining (NHEJ), which directly ligates the loose ends of DNA, often resulting in indels, or the high-fidelity homology direct repair (HDR), which requires a DNA template for recombination. Meganucleases, zinc finger nucleases (ZFNs), and transcriptional activator-like effector nucleases (TALENs) are examples of modified nucleases that are based on protein-DNA interactions and can target DNA in a sequence-specific manner for DSB purposes [31]. These proteins are engineered to recognize and bind DNA via covalent interactions between effector domains and short nucleotide sequences, then cut at these locations. The use of such site-specific nucleases for DNA or RNA cleavage offers a powerful toolkit for lncRNA investigation in vitro and in vivo [32]. However, nucleases driven by protein-DNA interactions are experimentally complex to engineer–for example, they require designing a string of zinc finger moieties that recognizes all possible trinucleotide combinations of the target sequence – and are highly expensive, limiting their range of applicability [32].

Further advancements in genome editing approaches arrived with CRISPR/Cas, a component of prokaryotic cells that functions as a protective mechanism against integrating viruses [33]. Several species of bacteria and archaea display an assortment of CRISPR/Cas components that differ in their mechanisms of action; still, the key features of the system remain the same: an RNA-guide (gRNA) sequence that drives a nuclease protein (Cas) to cleave nucleic acids [34]. After gRNA pairing, Cas proteins require the recognition of a nucleotide sequence for activity (a protospacer adjacent motif (PAM) upstream of the gRNA in type II, or a protospacer flanking sequence (PFS) in type VI systems) [35]. The user need only alter the sgRNA sequence to target DNA loci of interest. CRISPR/Cas is notably diverse in terms of structure and function, being generally divided into two classes according to the complexity of the protein effectors: multi-subunit effector complexes in class 1 or single protein effectors in class 2 [34]. From these, six types of Cas proteins can be structurally discriminated, and these can be further subdivided based on phylogeny. As the technology continues to evolve and new subtypes are identified, further classifications should emerge [36].

**Fig. 2:**
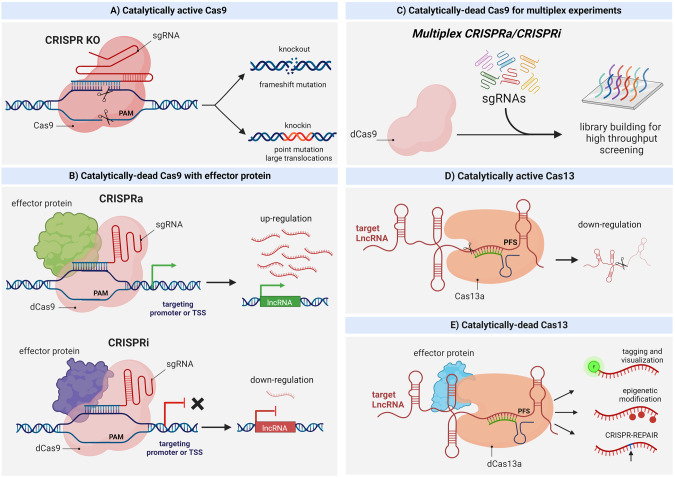
Summary of CRISPR/Cas tools. **A** CRISPR/Cas9 creates a double-strand break in a DNA region recognized by a sgRNA molecule. This results in frameshift mutations or knock-in mutations such as point mutations, where a DNA template is used for homology-directed repair. **B** Top: CRISPR activation (CRISPRa) utilizes a catalytically-dead (no nuclease activity) Cas9 molecule fused to an effector protein which acts as a transcriptional activator. Recognition of the target DNA sequence by sgRNA results in endogenous gene activation. Bottom: CRISPR interference (CRISPRi) follows the same approach with an inhibitory effector protein. **C** CRISPRa and CRISPRi can be used to generate gRNA libraries for high-throughput screening of ncRNAs. **D** CRISPR/Cas13 targets RNA for cleavage by recognizing a PFS sequence, resulting in the RNA’s downregulation. **E** A catalytically-dead Cas13 molecule can be used to localize effector proteins to RNA sites of interest for various applications, inclusing visualization by fluorescent probe tagging, epigenetic modification, and CRISPR-RNA Editing for Programmable A to I Replacement (REPAIR). Created with Biorender.

CRISPR/Cas approaches have been repurposed for a series of novel applications: genome editing, live cell tracking, epigenetic modifications, gene perturbation, etc. (Fig. 2) [37, 38]. The plethora of applications can be arranged into three types of experiments: (i) DNA/RNA manipulation generally with an active Cas protein, (ii) ‘recruitment’ experiments, which combine gRNAs with a Cas9 protein containing an inactive nuclease domain conjugated to an effector protein, and (iii) the modification of CRISPR/Cas systems for high-throughput screening and library construction [39]. CRISPR/Cas9, a class 2 type II system, is the most widely used and adapted CRISPR tool, and was designed and optimized for targeting mammalian cells [40]. This system contains a sgRNA comprising a 20 bp sequence complementary to the target region, a scaffolding and palindromic sequence (essential for Cas9 interaction), and the nuclease Cas9 (DNA binding protein responsible for DNA cleavage). The DSB occurs after sgRNA pairing and Cas9 recognition of the 5′ - NGG PAM sequence. The sgRNA and CRISPR/Cas9 technology have an expanding set of applications in neuroscience, constituting an easy and cheap method for targeting DNA [41].

To date, several online tools are available for sgRNA design to predict off-targets scores (for a systematic review, see Hanna and Doench [42]). Broadly speaking, traditional CRISPR/Cas9 can be used for attaining a frameshift mutation as a consequence of indels in the target genomic region or can be leveraged for point mutations or larger integrations with the combined used of a donor DNA sequence that is homologous to the targeted region [40]. In addition to ‘knockout’ approaches with only one sgRNA, gene editing can be programmed to accomplish larger deletions or chromosomal rearrangements by using multiple sgRNAs. For example, Han and colleagues used a dual sgRNA system to knockout a large lncRNA locus in mice [43], resulting in the irreversible elimination of the gene. A similar approach was used for a large elimination (>1000 bp) of the genomic region containing both alleles of the lncRNA *scaRNA2* in mice [44].

#### Applications of programmable nucleases to study lncRNA roles in neurodevelopment

CRISPR-based approaches can be beneficial for the functional interrogation of lncRNAs [45], including those that are tissue-specific and/or expressed at specific developmental stages. As an example, Allou and collaborators used CRISPR/Cas9 to engineer mouse stem cells bearing mutations on chromosome 2 and mimicking the genotype of limb malformation patients. Using this approach, the authors identified the lncRNA *Maenli* as involved in developmental defects during axis formation [46]. CRISPR-mediated knockout of the lncRNAs *DDX53* and *TUNA* in human stem cell-derived neurons has also been performed to assess effects on neurodevelopment and related dysfunction [47, 48]. In a more exploratory study of the brain, the lncRNA *Cyrano* was targeted by CRISPR/Cas9 to generate knockout transgenic mice and elucidate *Cyrano* function and its interaction with other ncRNAs during neural cell development [29]. Other studies have corroborated the roles of lncRNAs in establishing behavioral phenotypes in rodents using CRISPR/Cas9-mediated deletion of lncRNA expression in early stages of neural development [49, 50]. The in vivo dissection of putative functions and possible regulators of lncRNAs by CRISPR/Cas has benefited from proposed optimizations of plasmid engineering [51] and delivery [52], and CRISPR/Cas has been successfully delivered in post-mitotic neurons via intra-uterus injection [53] and nanocomplexes [54].

The future of genome editing is propelling CRISPR/Cas towards therapeutic approaches aimed at correcting neurodevelopmental defects [55]. For example, Angelman syndrome (AS), a severe neurodevelopmental disorder caused by an imprinting defect on the *UBE3A* gene, was successfully treated in fetal mice using CRISPR/Cas9-based gene therapy [56]. AS results from deletions or mutations on the maternally inherited *UBE3A* allele, while the paternally inherited allele is normally silenced by the lncRNA *UBE3A-ATS*. Wolter and colleagues used CRISPR/Cas9 to knockout the lncRNA in vivo, avoiding silencing of the *Ube3a* paternal allele and reestablishing endogenous levels of the Ube3a protein. While CRISPR/Cas9 knockout models have thus found utility in this neurodevelopmental application, in vivo applications should be monitored for effective delivery to target tissue(s), potential genotoxicity, immune reaction, and off-target nuclease activity.

#### Modifications of CRISPR/Cas and the interrogation of lncRNA function

Many advancements in CRISPR/Cas systems have been made since their inception, including the use of modified Cas9 proteins that are catalytically dead (dCas9). dCas9 retains its DNA binding ability without its nuclease activity. Moreover, dCas9 can directly or indirectly recruit effector domains to specific genomic loci, causing transcriptional interference, gene activation, (epi)genetic modifications (chromatin remodeling, hypermethylation, etc.), and more. dCas9 has been used to deploy lncRNA cargo to genomic and ectopic loci using a system called CRISPR-Display, allowing for investigation of lncRNAs in a way that separates their functions from the effects of their transcription [57]. Another recent adaptation of Cas9 comes from Cheng et al., who have developed a method to tag and manipulate lncRNA expression in vitro. The approach, called CRISPR-CTRL, utilizes a trap vector containing a puromycin selection cassette and an MS2 tagging sequence. Another plasmid expresses Cas9 and two gRNAs, one of which targets a genomic region regulating the target lncRNA’s expression, while the other targets the selection cassette for linearization of the gene trap vector. When localized at the transcriptional termination site of a lncRNA, CRISPR-CTRL inserts puromycin for selection purposes, which has also resulted in upregulation of the expression of several lncRNAs (*HOX, HOTAIR, TUG1, DICER1-AS, ZEB1-AS, MIAT*, and *PTENP1*). Meanwhile, targeted insertion of a synthetic poly-A signal carried by the trap vector into the transcription start site (TSS) of *TUG1* and *DICER1* has resulted in reduction of their expression [58]. The ability to easily engineer modifications in Cas proteins by removing catalytic activity, fusing to effector proteins, or combining with other techniques allows the design of more complex and functional studies of lncRNAs and could implicate their use in translational medicine.

#### Activation of lncRNA expression: the use of CRISPRa

It is often convenient to overexpress a gene to test for gain-of-function activity or to treat loss-of-function mutations with functional copies of a transcript involved in a compensatory pathway. In such lines of investigation, the mechanism of lncRNA action (*cis* vs *trans*) becomes relevant. *Cis-*acting lncRNAs tend to accumulate at the sites of their transcription, recruit regulatory complexes, and/or interact with genes nearby to their TSSs. By contrast, *trans-*acting lncRNAs travel to genomic sites distant to that of their transcription or even outside the nucleus [59]. Exogenous expression can be used to test for gain-of-function lncRNA activity; however, while exogenous expression is useful for supplementation of *trans-*acting lncRNAs, it may not reflect the function of *cis-*acting lncRNAs. Additionally, under exogenous expression, the transgene product must be localized and processed correctly, and, to emulate wild-type expression, the naturally occurring ratio of the transcript’s isoforms should be maintained during overexpression. In some cases, the process of transcription of a lncRNA locus is important for the function of the lncRNA [60], a consideration which cannot be accounted for by exogenous application of a plasmid. These stipulations suggest that endogenous gene activation is preferable for overexpression of lncRNA loci.

**Fig. 3:**
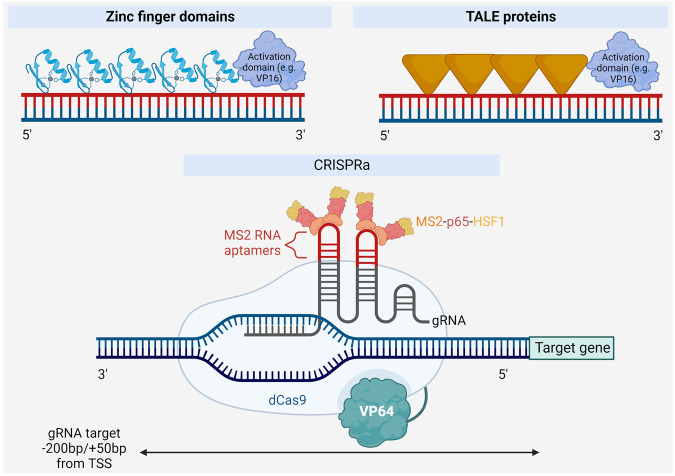
Methods for activating endogenous expression of a lncRNA. A carefully engineered combination of zinc finger domains or TALE proteins fused to a transcriptional activation domain can be designed to target the lncRNA promoter region. Alternatively, CRISPR activation (CRISPRa) utilizes a catalytically-dead Cas9 protein (dCas9) fused to a transcriptional activator and a gRNA with complementarity to a region near the transcription start site (TSS) of the lncRNA. The gRNA recruits two additional transcriptional activators, via its aptamers for robust transcriptional activation. Created with BioRender.

Targeted endogenous gene activation has been achieved with the use of several tools, particularly programmable nucleases (Fig. 3). The first transcription factors generated to endogenously activate gene expression were created by fusing zinc finger arrays to transcriptional activation domains such as the herpes simplex virion protein 16 (VP16) [61]. Similarly, approaches using TALE proteins fused to transactivation domains have been employed with varying degrees of efficacy. There is evidence that targeting TALE proteins to promoters can activate gene expression in high levels [62, 63]. TALE proteins can also be used in combination with steroid hormone receptor ligand-binding domains to allow conditional, on-demand gene activation in a robust manner [64]. However, both zinc finger and TALE proteins require the design and assembly of a series of new proteins for each target sequence, which requires knowledge of protein engineering and could be complicated and time-consuming.

Recently, modified CRISPR/Cas9 approaches have been used for targeted gene activation. CRISPR activation (CRISPRa) utilizes a dCas9 guided by a single RNA molecule to target a genomic region. The dCas9 protein is fused to transcriptional activation domains and thus facilitates transcriptional activation of the target gene [65–67]. In contrast to the zinc finger and TALE protein approaches, adaptation of this model to new target sites requires adjustment of just 20 base pairs, since the protospacer sequence is exchanged with the guide RNA cassette during targeting.

The use of CRISPRa to examine lncRNAs with prospective functional roles in the brain is relatively nascent. One recent advancement comes from Zhou et al., who have developed a method to activate multiple loci simultaneously in vivo in the nervous system. Improving upon the SunTag protein scaffold [68], which recruits several copies of an antibody-fusion protein (e.g. transcriptional activation domains) to a site of action (e.g. CRISPR/Cas9-delineated target site), the authors developed the SunTag-p65-HSF1 platform, which harnesses the p65-HSF1 fusion protein used in the synergistic activation mediator (SAM) system. SAM––which consists of the dCas9-VP64 fusion protein, a sgRNA, and the MS2-p65-HSF1 fusion transcriptional activator protein––displays robust activation capacity in Neuro-2a (N2a) mouse neuroblastoma cells [66]. Zhou and colleagues found that SunTag-p65-HSF1 (SPH) activated endogenous transcription factors in N2a cells and in primary mouse astrocytes with gRNAs designed to target regions upstream of the TSS of these genes. The off-target activity of the fusion protein was determined to be minimal [69]. For application to multiple loci, including lncRNA loci, the authors expressed SPH as an inducible transgene in murine brains and, by delivering sgRNA complexes, successfully activated combinations of four lncRNAs, *Miat, Halgr, Fendrr* and *Lncpint* in neural tissue [69]. The ability to activate multiple lncRNA loci in vivo at once could prove useful to the interrogation of lncRNA synergism and/or interactions. This approach is particularly potent in the context of modeling neurodevelopmental disorders, which can be genetically complex or heterogeneous, requiring precise interrogation of multiple genes at once.

An area of interest in the study of lncRNA function in the CNS is the ability to assay changes in epigenetic modifications induced by the transcript, which can control temporospatial expression of neurodevelopmental and other genes [70]. Many lncRNAs – such as the large intervening ncRNA *HOTAIR*, which is crucial for cell growth and viability–can interact with chromatin-modifying complexes and drive them to specific genomic loci, often resulting in changes in gene expression [71–73]. The nuclear-enriched lncRNA *NEAT1* is alternatively spliced into two isoforms and has been observed to scaffold chromatin-modifying proteins and to bind genomic loci directly [74]. As *NEAT1* is enriched in glia of the mammalian brain, is linked to neuroplasticity and neurodegeneration [75, 76], and has been shown to interact with components of the H3K9me2 methyltransferase complex in neurons [77], its role in the CNS is of interest, particularly in vivo. Using CRISPRa, Butler et al. have investigated the role of *NEAT1* in H3K9me2 activity in the aging rat hippocampus, finding that overexpression of the transcript enhanced histone methylation and *c-FOS* repression. Overexpression of *NEAT1* also induced impairments in hippocampus-dependent memory formation [77]. In this approach, unlike in SAM, the dCas9 protein is fused to two copies of the VP64 transactivation domain and bilaterally injected, along with a sgRNA expression vector, into CA1 of the mouse hippocampus. While the transcriptional and behavioral outcomes were significant, the approximately two-fold increase in *NEAT1* expression using this approach was lower than the roughly ten-fold change found by Zhou et al., suggesting that the efficacy of CRISPRa in vivo should be titrated by adjusting the number and variety of gRNAs and transactivation domains used.

#### Challenges associated with CRISPRa in the study of lncRNAs

**Fig. 4:**
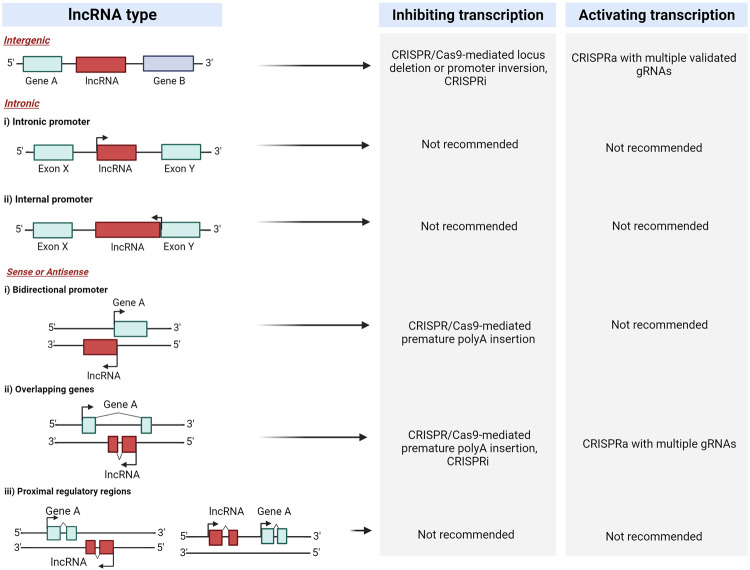
Recommended methods for genome editing of lncRNA loci. Diagrams on the left depict common instances of lncRNAs with varying genomic origins relative to their neighboring genes. The recommended approaches for targeting each lncRNA to achieve specific inhibition or activation are listed on the right. In some scenarios, no suitable approach is currently available. Created with BioRender.

The CRISPRa system is an advantageous method of lncRNA activation in which sgRNAs are targeted upstream of the promoter or TSS of a lncRNA gene and recruit dCas9 fused to one or more transcriptional activation domains. This approach maintains endogenous mechanisms of transcription and post-transcriptional processing while avoiding disruption of the genomic locus. A common concern with CRISPR/Cas9 approaches is off-target effects; indeed, gRNAs designed for CRISPRa-mediated lncRNA induction have been shown to recognize off-target binding sites with low bioinformatic predictability [78]. Additionally, many lncRNA loci are derived from bidirectional promoters, overlap with genes located in the lncRNA intronic region, or contain promoters that lie within the gene bodies of coding genes. Given these architectural complications, Goyal et al. conducted a study of all annotated coding and noncoding genes in the human genome to identify those lncRNA loci that are “CRISPRable”. For effective CRISPR-mediated activation, these loci must not be: i) bidirectional (derived from promoters situated within 2000 bp upstream or downstream of another promoter), ii) transcribed from internal promoters (those promoters that lie within the gene bodies of other genes), or iii) transcribed from promoters which regulate other genes. Using these rules, Goyal et al. found that, of nearly 16,000 lncRNA loci investigated, only 38% were suited for CRISPR approaches and would not interfere with expression of neighboring genes [79]. Care must be taken to ensure that the lncRNA locus in question can be targeted using sgRNAs with high on-target specificity and low likelihood of perturbing neighboring loci. Measures to ensure that expression of surrounding genes is not being affected throughout the experiment should also be taken. Recently, a manually curated database of validated paired gRNAs for targeting lncRNA loci has been created by Chen et al. to assist in this endeavor [80]. We provide recommendations regarding genome editing (ablation and activation) of lncRNA loci in Fig. 4.

Another consideration of the CRISPRa approach is its transience. Unlike gene editing events that incorporate elements directly into DNA, inducing expression via CRISPRa is detectable in the highest proportion of cells shortly after the time of introduction of the gRNA and dCas9 plasmids. These considerations make amplification and generation of lines stably expressing the activated gene difficult. One approach to address this issue could be to knock-in the dCas9-transactivation domain and sgRNA cassettes under control of a constitutively active promoter such as CAGGS; a similar approach was previously used to drive transgenic red fluorescent protein expression to monitor miRNA activity [81, 82]. Additionally, transduction of a lentivirus-packaged plasmid increases efficiency and duration of expression compared to direct transfection of the expression vector [83, 84]. Cell lines that are stably transduced with the SAM components can be purchased or generated and transduced or transfected with the gRNA vector. The viability of these techniques in vivo in the CNS depends on effective delivery, minimal toxicity, and high cell-type specificity; successful in vivo overexpression of some lncRNAs (Table 1) points to their potential as therapeutic targets in the CNS [85].

**Table 1:** In vivo targeting of lncRNAs for activation in the CNS over the past five years.

LncRNA(s) Targeted	Approach	Approx. Fold Change in target tissue in vivo	Outcome	Reference
***Miat, Halgr, Fendrr, Lncpint***	Cre-dependent SPH transgenic mouse stereotactically injected (cerebral and cerebellar cortices) with dual AAV containing Cre and a combination of gRNAs for each lncRNA target	3 to 4	Proof of concept	[69]
***NEAT1***	Bilateral intra-CA1 injection of lentiviral package containing dCas9-VP64-VP64 and gRNA targeting *NEAT1* promoter region in mouse	2	Impaired memory formation in contextual fear conditioning model	[77]
***NEAT1***	Mouse intracerebroventricular injection of adenovirus expressing the short isoform, *NEAT1_1*	2.7	Decreased neuronal apoptosis and improved motor function, spatial memory, and learning ability after TBI	[164]
***lncRNA-N1LR***	Mouse cerebral cortical injection of adenoviral vector containing full-length mouse cDNA *lncRNA-N1LR* sequence	3.5	Reduced neuronal apoptosis and infarct volume after middle cerebral artery occlusion	[165]
***PVT1***	Mouse lateral ventricle injection of lentivirus expressing *PVT1*	2.7	Increased tissue iron and malondialdehyde (indicators of cerebral ischemia)	[166]
***BCYRN1***	Tumor xenograft mouse subcutaneously injected with U251 glioma cells containing a lentivirus expressing full-length *BCYRN1*	Not specified	40% reduction in glioma volume	[167]
***LncRNA-1810034E14Rik***	Mouse cortical injection of lentivirus expressing *lncRNA-1810034E14Rik*	7	Decreased infarct volume in middle cerebral artery occlusion model, reduced inflammatory cytokines, microglial cell activation, and p65 phosphorylation in ischemic stroke model	[168]
***FOXD1-AS1***	Subcutaneous mouse flank injection of human glioma cells (Hs683) expressing vector containing full-length, wild-type *FOXD1-AS1*	Not specified	Increased tumor volume and weight, more rapid development	[169]
***TCONS_00004099***	Zebrafish xenograft by yolk sac embryonic injection of human glioma cells (U251 and U87) transfected with lentivirus vector packaged with TCONS_00004099 overexpression sequence	Not specified	Increased tumor migration and invasion	[170]
***MIAT***	Rat cortical injection of lentivirus expressing *MIAT*	3	Improved motor function, decreased miR-211 expression, and increased GDNF expression in striatal tissues of neonatal model of hypoxic-ischemic injury	[171]
***NEAT1_1***	*Drosophila melanogaster* overexpressing transgenic human NEAT1_1 in retinal photoreceptor neurons under control of the GMR-GAL4 driver using GAL4-UAS	1.5 to 2.5	NEAT1_1 transgenic expression improves retinal thinning and retinopathies in TDP-43-overexpressing fly models of ALS in a dose-dependent manner	[172]
***H19***	Mouse midbrain injection of lentivirus-packaged *H19* in Parkinson’s disease model	25	Inhibition of dopaminergic neuronal apoptosis, increased tyrosine hydroxylase production in substantia nigra pars compacta, improved motor ability	[173]
***lncRNA 4344***	Rat intracerebroventricular injection of lentivirally-packaged *lncRNA 4344*	6	Increased cognitive impairment, hippocampal neuronal apoptosis, and inflammatory response in rat model of cognitive impairment	[174]
***ARST***	Human glioma cell line (GL261) transfected with lentivirally-packaged *ARST* intracranially implanted in mouse	Not specified	Decreased glioma formation, improved survival	[175]
***MIR4435-2HG***	Subcutaneous flank injection of human GBM cells expressing *MIR4435-2HG* in mouse	Not specified	Accelerated tumor growth	[176]
***RIAN***	Mouse cerebral injection of AAV expressing *RIAN*	Not specified	Decreased reactive oxygen species production, cerebral infarct volume, and cell death in ischemia-reperfusion injury model	[177]

### Transcript-level manipulation

#### Overview of post-transcriptional methods for loss-of-function lncRNA experiments and applications to neurodevelopment

Although genome editing can shed light onto lncRNA function, in some cases, genomic targeting might interfere with the stability and expression of the host genome, obfuscating the ability to discriminate gene-regulatory roles performed by ncRNAs, the functions of the DNA encoding the lncRNA, or the act of ncRNA transcription. Moreover, genome editing is virtually irreversible, while transcript targeting offers temporary disruptions and thus a different set of possibilities for ncRNAs studies [86]. Three major approaches are available for direct targeting of RNA for degradation: RNA interference (RNAi), antisense oligonucleotides (ASOs) and, most recently, CRISPR/Cas13 technology. These methods are powerful tools to assess disease mechanisms and therapeutic interventions that can be directly mediated by lncRNAs.

RNAi consists of designing a small RNA molecule that complements the targeted RNA, triggering its degradation via the RNA-induced silencing complex (RISC). RNAi is an efficient, cheap, and quickly designable technique for RNA knockdown, but it is prone to off-target effects [87]. ASOs are small, single-stranded sequences of DNA (8–50 base pairs in length) that form DNA:RNA chimeras, leading to RNA degradation via RNAse H or steric ligation, which blocks splice sites [88, 89]. An advantage of ASOs in RNA targeting is their localization to the nucleus and ability to trigger RNaseH activity for robust lncRNA cleavage [90]. However, ncRNAs are flexible molecules that can assume a variety of conformations [91], which complicates structure predictions for proper ASO targeting design.

Both RNAi and ASOs account for most of the evidence concerning lncRNA function in cell differentiation/commitment and organismal development, mostly derived from loss-of-function experiments. Recently, these technologies have been applied for in vivo clinical purposes as means of gene therapy [92–95]. However, the use of ASOs and RNAi can not distinguish between *cis-* and *trans-*acting lncRNAs, and there are considerations for their application to lncRNA targeting given that (i) the RISC complex that mediates RNAi mechanisms is predominantly cytoplasmic, while many lncRNAs are localized in the nucleus, (ii) those lncRNAs that are expressed abundantly may not be targeted completely by RNAi or ASOs [96], and (iii) the effects of these silencing techniques are transient, complicating long-term functional studies of the target lncRNA. Furthermore, in vivo delivery of RNA-based tools can be complicated by high immunogenicity and the challenges of targeting the molecule to the lncRNA-expressing cell type [97].

Recent approaches using aptamers (short––up to 200 nucleotides––single-stranded oligonucleotides that, much like antibodies, form secondary hairpin and tertiary structures that can recognize a specific molecular target in its 3D conformation) for direct lncRNA targeting show benefits that RNAi and ASOs alone do not, including low immunogenicity and high in vivo penetrance and stability [98–100]. Aptamers can be constructed to target lncRNA and/or lncRNA-protein structures for localization, isolation, and direct functional investigation. Chimeric aptamers have also been used to deliver RNAi to lncRNA molecules in vitro via recognition of and interaction with lncRNA regulatory proteins [101]; however, it appears that the chimeric aptamer approach for lncRNA targeting has not yet been implemented in vivo in the CNS. While design of such aptamers must be carefully planned to ensure correct folding and avoidance of structural perturbations to the lncRNA target upon binding, it will be interesting to monitor the progress of these tools in neurodevelopmental studies, given that the small size (<25 kDa), relatively low production cost, reduced immunogenicity and toxicity, and stability over a variety of pHs [102] make aptamers a good option for nanoparticle-mediated delivery across the BBB. Indeed, aptamer-drug nanoparticle conjugates, which have gained ground in targeted cancer therapeutics [103], could pose exciting new options for neurodevelopmental disease therapies.

Novel CRISPR/Cas technologies are emerging and offer a variety of alternative possibilities for post-transcriptional targeting. The first attempt to directly target RNA with CRISPR/Cas technology used the CRISPR/Cas9 tool; in fact, initially this nuclease showed an affinity to RNA molecules [104]. The technology was later optimized for this purpose by using a co-delivery system of sgRNA with another construct: a short duplex oligonucleotide that served as an exogenous PAM sequence, named PAMmer [105]. While some Cas9 orthologs appear to have RNAse activity without the need for a PAMer in bacteria [106], mammalian cells require an exogenous PAM for targeting RNA, and the need for these synthetic oligos hinders the use of this method in large pooled screenings.

Ultimately, with the discovery of class 2 type IV CRISPR effectors (CRISPR/Cas13 or C2p2), RNA targeting by CRISPR/Cas was optimized and presented several advantages, including the fact that it was RNA-guided, highly efficient, easy to design, and had reduced off-target effects. In this system, a simplified gRNA binds to the Cas13 nuclease, which recognizes a protospacer flanking sequence (PFS) on the target RNA without the need for synthetic PAMers [107]. After CRISPR/Cas13 assembly with the target RNA molecule, Cas13 actively cleaves the RNA, leading to its knockdown. Like other CRISPR/Cas machinery, several members of the Cas13 family have been described, each containing different structural effector domains (gRNA) and degrees of affinity/activity [108]. In a recent paper, Zhou and colleagues developed a method for disrupting the mRNA of an RNA-binding protein, Ptbp1, using an ortholog of the Cas13 family (Cas13d or CasRx). CasRx is a smaller and easier-to-pack variation of the CRISPR/Cas13 system that can be delivered via subretinal injection of an adeno-associated virus (AAV) package. Knockdown of *Ptbp1* transcript using this system successfully converted Müller glia to retinal neurons [109]. Li and colleagues have also used neuron-specific promoter-driven expression of a high-fidelity CRISPR/Cas13 system to suppress *UBE3A-ATS* for the treatment of Angelman Syndrome in a murine model, showing the potential applications of this system to neurodevelopmental studies [110].

Cas13 can also be exploited to function as a recruitment protein. Zhang et al. have optimized CRISPR/Cas13 for RNA editing in mammalian cells using a catalytically dead Cas13 fused to the adenosine deaminases acting on RNA (ADAR2) enzyme. The strategy, named REPAIR (RNA Editing for Programmable A to I Replacement), facilitates the exchange of adenosine to inosine [111], an intrinsic post-transcriptional modification that is capable of altering the sequence of RNA molecules [112]. This strategy opens new possibilities for interrogating lncRNA functional modifications, especially considering that post-transcriptional modifications such as N6-methyladenosine (m6A) are particularly enriched in the developing brain [113]. Epitranscriptome analysis shows an array of m^6^A alterations in many neurological disorders, though the biological functions of m^6^A-modified lncRNAs in the brain remain elusive. The REPAIR system thus holds potential to address questions in neurodevelopmental epigenetic and ncRNA biology.

#### Ectopic expression of lncRNAs and recent applications in neurodevelopmental studies

Important to validating the results of deletion or knockdown of lncRNA expression is the ability to restore expression to endogenous levels and observe whether phenotypes of interest are “rescued” or not. In the case of lncRNAs, exogenous rescue can help determine whether the RNA is *cis-* or *trans-*acting. While many *cis-*acting lncRNAs have been identified and investigated, the modes by which *trans-*acting lncRNAs act upon chromatin and distal genes are less understood [19, 21]. Ectopically expressing a lncRNA in *trans* after its deletion can help decipher whether the endogenous lncRNA acts in *cis* and elucidate its mechanisms of action.

Traditionally, exogenous expression of a gene of interest involved pronuclear injection of the cDNA sequence under control of a strong exogenous promoter. However, this approach foregoes the inclusion of endogenous regulatory sequences (e.g. enhancers, insulators, etc.) and restricts the size of the gene that may be injected. With the advent of yeast and bacterial artificial chromosomes (BACs), cloning capacity increased and lncRNAs such as the 16.5-kb human X inactivation-specific transcript, *XIST*, saw successful in vivo mammalian transgenesis [114]. Transgenics can also be carried out using RNA viral vectors like recombinant retroviruses, lentiviruses, or DNA viral vectors like AAVs. These vectors are advantageous in ectopic rescue experiments not only because they recapitulate native expression of the lncRNA (under control of its endogenous promoter and regulatory elements) but also because they incorporate the transgene into the host genome for stable expression. By contrast, pronuclear delivery of the transgene by non-viral methods achieves transient expression.

Ectopically expressing lncRNAs using BACs has led to insights regarding whether they act in *cis* or *trans* to mediate mammalian neurodevelopment. One example comes from work by Andersen et al. on *Pnky*, an evolutionarily conserved nuclear lncRNA that interacts with the mRNA splicing regulator PTBP1 to mediate neurogenesis and neuronal differentiation [115]. *Pnky* is transcribed divergently (in the opposite direction) from *POU3F2*, a neighboring coding gene whose product serves as a proneural transcription factor. Conditional deletion of murine *Pnky* resulted in enhanced neurogenesis [116] and did not affect *Pou3f2* expression, suggesting that *Pnky* does not act in *cis* [117, 118]. Ectopic expression of a BAC construct containing 170 kb of the genomic sequence surrounding *Pnky* but lacking the *Pou3f2* coding sequence rescued neurogenesis, suggesting that ectopic expression of *Pnky* in *trans* is sufficient to rescue endogenous *Pnky* deletion phenotypes [116]. Elucidating this mechanism of action can be useful for understanding the contribution of lncRNAs to neurological disorders and the potential for lncRNA gene therapy.

**Fig. 5:**
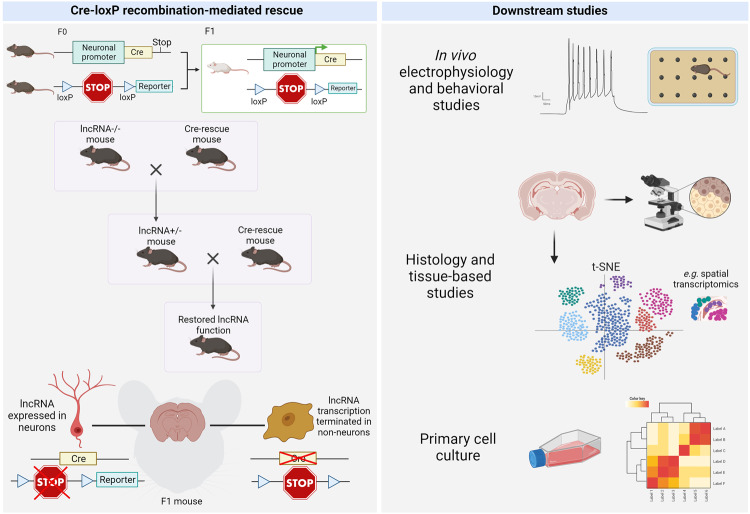
Cre-loxP recombination-mediated gene rescue and downstream studies. (Left) Cre-lox recombination system for rescuing expression of lncRNAs in vivo. A cell-type-specific promoter is used to drive expression of Cre in a lncRNA-null mouse expressing a premature stop cassette flanked by loxP sites. The premature stop is thus removed to restore endogenous expression of the lncRNA. Target cells will thus have restored expression of the lncRNA, while non -target cells will maintain the truncation. (Right) In vivo (e.g. behavioral studies), tissue-based (*e.g*. histology or spatial transcriptomics), and/or in vitro assays (e.g. single-cell RNA sequencing) can be performed to confirm physiological restoration of lncRNA downstream functions. Created with BioRender.

In vivo studies of ectopically expressed lncRNAs in the mammalian brain often take the Cre-recombination transgenic approach (Fig. 5). There are challenges, however, in targeting exogenous lncRNA expression to the correct cell type. This issue has been addressed by Cajigas et al. in a study of the ultraconserved enhancer lncRNA *Evf2*, which serves as a transcriptional coactivator of members of the Dlx/dll homeodomain-containing family. *Evf2* can be alternatively spliced to function simultaneously as a *trans-*acting activator and *cis-*acting repressor to induce changes in chromosome topology and gene expression [119]. To distinguish between *Evf2 trans* and *cis* mechanisms, Cajigas et al. rescued biallelic expression of a loss-of-function *Evf2* model using Cre-mediated recombination and an enhancer that specifies interneuron differentiation and migration. Long-range *trans* rescue expression of *Evf2* in the developing mammalian forebrain was ultimately found to modulate interneuron diversity and adult seizure susceptibility [119].

Similar approaches have been taken to express *FIRRE* lncRNA in mouse common lymphoid progenitors [120] and either of the two isoforms of the lncRNA *NEAT1* in mouse embryonic fibroblasts (MEFs) [121]. *FIRRE* is encoded by an X-linked gene and enacts changes in autosomal gene expression with roles in hematopoiesis. *NEAT1* is a nuclear lncRNA necessary for the formation of subnuclear bodies called paraspeckles, which harbor proteins and RNA capable of cellular and chromatin modifications that form near the transcription start site (TSS) of the *NEAT1* gene [122]. Transgenic rescue of these genes has revealed that, though the endogenous copy acts near the transcription site, exogenous *trans* expression is sufficient to restore lncRNA function. While these methods seem to parse whether predicted *cis-*acting lncRNAs are truly *cis-*acting, some important considerations must be made. First, the transgene should be integrated into a chromosome different than that of the endogenous locus – this can be verified by testing for gene linkage in offspring of the desired crosses. Additionally, successful rescue in *trans* depends on timing and expression levels of the gene, so appropriate measures should be taken to ensure that the lncRNA is rescued to physiological levels corresponding to the developmental state of the cell type in question.

There are other foreseeable challenges regarding methods of ectopic expression. In the case of Cajigas et al., the *Evf2* transgene rescued just 38% of wild-type *Evf2* expression, suggesting that some transgenes might be susceptible to dosage deficiencies. Additionally, large lncRNAs may be difficult to transfect, as the expression vector may permit only a limited insert length. Indeed, larger constructs can be more cell toxic [123]. Lastly, once expressed, the lncRNA must be correctly localized (for example, in the nucleus versus the cytoplasm), processed, and folded to function correctly [124, 125]. Localization should be verified using techniques like RNA FISH, while polyadenylation and 5′ capping can be examined by RNA-seq [126] and monitoring guanine-N7 methyltransferase activity using fluorescent probes [127], respectively.

### Special considerations: circRNAs, CNS delivery methods of ncRNA tools, and functional screens of lncRNAs

#### circRNAs: an overview

LncRNAs may be expressed in linear or circular form, the latter comprising a class of ncRNA called circRNAs. CircRNA expression is particularly enriched in the brain, plays a regulatory role in neurogenesis and synapse formation/maintenance [128], and may be dysregulated in neurodevelopmental and psychological conditions [25]. Although the existence of circRNAs has been known for years, their mostly cytoplasmic presence in eukaryotic cells was long attributed to incorrect RNA folding and possible artefacts. Only with the expansion of next-generation technologies did circRNA detection––which occurs once bulk RNA is depleted of linear and ribosomal RNAs––and later functional characterization improve [129]. While relatively unabundant, over 100,000 unique circRNA molecules have been reported in humans [130], and around 30% of transcribed genes in the brain have the potential to produce circRNAs [131]. However, the majority of circRNAs are still understudied. Traditionally, they are suggested to control gene expression by acting as ‘sponges’ for different miRNAs. In addition, as circRNAs are very stable, their slow turnover and cell type-specific and/or tissue-specific expression suggest they have great potential as disease biomarkers [132].

#### Loss-of-function circRNA manipulation

circRNAs are covalently circularized molecules produced by back-splicing, a non-canonical event that results in the circularization of specific exons or/and introns [133]. Most circRNAs are formed by one or multiple exons, normally overlapping with coding genes, where the back-splicing mechanism competes with traditional splicing from the host gene (Fig. 1). As a result, two important issues are involved in manipulating circRNA expression: first, at the genome level, it is difficult to target circRNAs without affecting expression of the host gene, and second, at the transcript level, circRNA expression can compete with its linear RNA counterpart during splicing production. As such, targeting circRNAs can affect the balance of backsplicing over canonical splicing [134]. To overcome these issues, the best alternative available is to target the circRNA transcript directly at the circularized junction [135]. Direct circRNA targeting avoids disrupting the gene locus and consequently the neighboring coding gene. Additionally, considering most circRNAs are exonic and share their sequence with their coding gene counterpart, targeting the circularized region ensures specific targeting of the circRNA without disrupting the mRNA of the host gene [136]. Few cases of targeted circRNA knockdown have been reported in mammalian CNS models with the goal of studying development. One recent study by Suenkel et al. used siRNA targeted to the head-to-tail junction of *circSLC45A4*, which is expressed in high levels in human embryonic frontal cortex, in a human neuronal cell line to demonstrate that the circRNA is required to maintain neural cells in a progenitor state [137]. At the genomic level, Piwecka and colleagues have used CRISPR/Cas9 to knock out the murine circRNA *Cdr1as* gene locus in vivo. *Cdr1as* is highly efficiently circularized and cannot be detected as a linear transcript [138]; in such cases a genome editing approach may be efficient.

#### Overexpressing circRNAs

Ectopic expression and endogenous activation of circRNAs is difficult for many reasons. The biogenesis of circRNAs is not fully understood, and attempts to overexpress circRNAs in vitro with or without neighboring introns have led to linear byproducts [137]. In theory, mammalian vectors comprising the circularized exon, flanking splice signals, and intronic sequences having inverted repeats that allow for backsplicing and circularization can be generated; however, linearization can still occur if RNA polymerase bypasses transcription termination signals and the vector is transcribed in a rolling-circle manner, leading to a product with exon repeats that undergoes canonical splicing and yields multiple undesired transcripts [139]. Still, circularization can be induced using the bacterial endoribonuclease Csy4, a member of the CRISPR family. Csy4 cleaves at the 3′ base of a 16-ribonucleotide hairpin, leaving a shortened sequence to generate crisprRNAs that will recognize a target CRISPR sequence [140]. Csy4 has been used to induce circularization of GFP pre-mRNA in mammalian cells by intron cleavage and removal of completing splice signals, which results in circularization on par with the endogenous process [141]. In vivo examples of circRNA activation in the CNS are few, however Bai et al. have introduced *circDLGAP4*, a miRNA sponge whose expression is decreased in stroke patients, via microinjection in the lateral ventricle of a mouse stroke model [142]. This approach reduced neurological deficits and infarct areas in mouse transient middle cerebral artery occlusion models. It appears that neither in vitro nor in vivo CRISPRa of circRNA loci in the CNS has been conducted as of yet; investigating this technique (i.e. which region of the endogenous gene to target, the number of gRNAs required for effective activation, efficient methods of delivery of CRISPRa components, etc.) could result in a useful tool for further circRNA functional studies.

#### Addressing in vivo delivery methods to the CNS

Many technical challenges and limitations hamper the in vivo investigation of lncRNAs in the brain, including the fact that patient brain samples are not readily available for biopsies and are normally variable (age, sex, etc.), and that lncRNA expression might be highly specific. Currently, transgenic mammalian models, namely rodents, are among the major applications of in vivo lncRNA editing tools like CRISPR/Cas [143]. Recently, Perry and colleagues used a transgenic mouse generated with CRISPR/Cas9 and CRISPRa to knockdown and activate the lncRNAs *Silc1* and *Norris* in neural cell types [144], showing their role in normal neural development and later in neurodegenerative processes. For precise temporal control of expression (rather than inducing genetic changes in embryo), post-transcriptional targeting can be used (e.g., RNAi). Another major hurdle is in vivo delivery of any genetic tool to a mature brain, especially in cases in which the target is not the whole tissue but particularly cellular subtypes. Most approaches involve either viral particle infection or direct plasmid-based delivery. In the case of CRISPR/Cas approaches targeting lncRNAs, several studies have proposed optimizations of plasmid engineering [51] and delivery [52, 145].

**Fig. 6:**
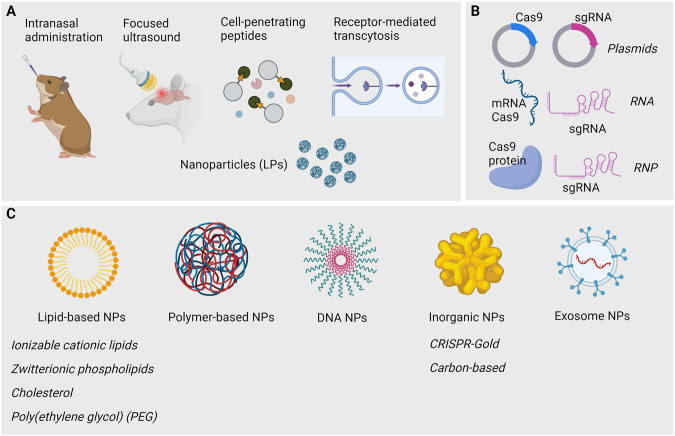
Summary of non-invasive methods for delivering CRISPR/Cas9 tools across the BBB. **A** Non-invasive methods include intranasal delivery, focused ultrasound, cell-penetrating peptides, receptor-mediated transport, and nanoparticle-based carriers. **B** CRISPR/Cas9 delivery can take the form of a plasmid, as RNA for Cas9 mRNA and sgRNA, or Cas9/sgRNA ribonucleoprotein complexes (RNP). **C** Various types of nanoparticles are under development, including lipid or polymer-based structures, DNA or inorganic nanostructures, as well as exosomes. Created with BioRender.

The BBB poses a particular limitation on CRISPR/Cas plasmid delivery to the brain. Various strategies are employed to enhance BBB permeation. BBB traversal depends on the transport route (e.g., paracellular, intracellular) and the nature of engineered particles (e.g., lipid-based, organic-based). These strategies can be categorized as invasive – including methods like direct brain injection, intrathecal delivery, intracerebral grafts, and deep brain stimulation––or non-invasive–– encompassing nanoparticle-based carriers, biological mechanisms (e.g., cell-penetrating peptides, receptor-mediated transport), focused ultrasound, and intranasal delivery [146] (Fig. 6A). Currently, a few nanocarriers are designed to transport both the Cas9 protein and sgRNA safely across the BBB; these are discussed below. Moreover, the Cas9 protein can be delivered in three modes: as a plasmid, as mRNA, or as a protein [147]. In each case, distinct carriers can be modulated to achieve effective transport, target-specific delivery, and endosomal release for cytoplasmic function (Fig. 6B).

Lipid-based nanoparticles (LNPs) are the classic and most common form of nanoparticle delivery; unfortunately, LNPs packaged with CRISPR/Cas9 plasmids have a very low efficiency in vivo and thus require optimizations to improve their stability and their target specificity. One alternative to LNPs is engineered and synthetic zwitterionic amino lipids, which have demonstrated efficient (co)delivery of Cas9 mRNA and sgRNAs in vivo and in vitro [148]. Polymer-based carriers are another alternative for CRISPR/Cas9 delivery, serving as chemically diverse molecules that have great potential for target-specific approaches and endosomal escape [149]. Other options include DNA nanostructures, which are self-organizing DNA complexes that offer a large loading capacity and reduced toxicity [150], and gold nanoparticles, which are functionalized particles that allow laser-controlled release of the CRISPR/Cas9 system. This CRISPR-Gold approach has shown great promise to target neurological disorders [151]. Finally, exosomes are versatile membrane-bound vesicles that offer major advantages in terms of biocompatibility and encapsulation of cargo, but to date, they have mostly been used to deliver small ncRNAs (such as siRNAs and miRNAs) [152] (Fig. 6C). A variety of innovative approaches are being explored to enhance the efficiency and specificity of CRISPR/Cas9 delivery across barriers such as the BBB; still, the safety of patient-facing gene therapy and the possibility of cell type-specific therapies while targeting the brain remain active areas of research.

#### Functional screening and selection of candidate lncRNAs in disease contexts

High-throughput forward genetic screening of lncRNA loci is a powerful tool for the selection of candidate disease-causing genes. Tools to endogenously ablate lncRNA expression can prove useful for screening samples over time; indeed, many studies have been conducted using CRISPR/Cas9 and CRISPR/Cas13 for this purpose. For example, Zhu et al. have developed a paired gRNA approach in which two gRNAs are introduced under the control of two separate promoters in a single vector [153]. Using a paired sgRNA library targeting the promoter and/or promoter plus exon regions of 671 lncRNA genes with putative oncogenic roles, Zhu et al. found 51 lncRNA genes that regulate cancer cell growth. Important considerations in this method included: (i) filtering sgRNAs for predicted efficiency scores [154], relatively low GC content, and mismatches to other loci in the genome and (ii) ensuring that sgRNAs had the same transcriptional orientation as the target lncRNA and excluded exonic regions of neighboring coding genes.

A similar approach can be taken to delete lncRNA expression by targeting sequences −50 bp to +75 bp surrounding 5′ splice donor and −50 bp to +75 bp surrounding 3′ splice acceptor sites. Unlike promoter targeting, which disrupts the process of transcription, splice site targeting can induce exon skipping or intron retention and disrupt the maturation of the RNA [155]. In some cell lines, splice site-targeting sgRNAs outperform exon-targeting sgRNAs in effectively disrupting expression [156]. Meanwhile, screens using post-transcriptional knockdown such as RNAi, CRISPRi (which utilizes the dCas9 protein fused to a repressive KRAB domain, thus serving as a transcriptional ‘roadblock’), or CRISPR/Cas13 can be effective tools for functional characterization of lncRNAs; however, these tools are limited by the efficiency of the knockdown and specificity to the target transcript and are transient interferences [157, 158]. Another consideration in genetic screening is the influence of the chromatin state on predicted DNA-protein, RNA-DNA, and RNA-RNA interactions. For example, genes with a higher percentage of euchromatin could appear more frequently as functional candidates than their counterparts, which may still carry functional implications. Similar limitations apply to forward genetic screens using CRISPRa, which has been used in a few high throughput screens of lncRNAs in the CNS [83, 159]. Generation of CRISPR-, CRISPRi-, and CRISPRa-mediated lncRNA libraries for genome-wide screening should be conducted to expand annotation of lncRNAs associated with CNS and associated developmental processes.

#### Concluding Remarks

LncRNAs are changing the way we think about cellular functions. From gene expression regulation to structural safeguards, a variety of functional noncoding transcripts are emerging as key effector molecules in many cellular programs. The particular abundance of lncRNAs in the brain provides them with special appeal for the neurobiologist, not only from the mechanistic point of view but also in terms of their potential as targets and/or biomarkers in a number of CNS pathologies. The development of approaches that allow detailed characterization of lncRNAs in the CNS and in CNS pathologies is therefore pivotal for a better understanding of their roles. In this review we have described techniques for lncRNA analysis, with an emphasis on CRISPR/Cas systems and applications to experimental neurodevelopmental biology. These techniques are summarized in Table 2. The growing number of functional lncRNAs identified in neural development and/or maintenance warrants further exploration of this type of molecule. However, despite prominent advances over the last years, there are still important caveats that hinder the study of lncRNAs in the brain.

**Table 2:** Summary of approaches used to modulate lncRNA expression.

	CRISPR/Cas9	CRISPRa	Exogenous expression	CRISPRi	CRISPR/Cas13	REPAIR	ZFNs and TALENs	RNAi and ASOs
**Catalytic actor**	Active Cas9	Inactive Cas9	N/A	Inactive Cas9	Active Cas13	Inactive Cas13	ZFN and TALEN	RISC and RNAseH
**Molecular target**	DNA	DNA	N/A	DNA	RNA	RNA	DNA	RNA
**Outcome**	DNA editing	Transcription modulation	Expression of part- or full-length transcript	Transcription modulation	RNA editing	RNA modification	DNA editing or transcription modulation	RNA modulation
**Effector protein**	Cas9	Multiple	N/A	Multiple	Cas13	ADAR2	Multiple	N/A
**In vivoexamples in CNS cited in this text**	*Cyrano* [29]*, AtLAS* [49]*, Ube3a-ATS* [56]	*Miat, Halgr, Fendrr and Lncpint* [69]*, for more, see* Table 1	*Pnky* [116]*, Neat1* [164]*, for more, see* Table 1	N/A	N/A	N/A	N/A	*Pnky* [95]*, Ube3a-ATS* [94]
**Multiplex possibility**	YES	YES	NO	YES	YES	-	Limited	YES
**Advantages**	Stable expression of mutant, technically feasible, economical	Robust transcriptional activation while maintaining endogenous mechanisms, no permanent alteration to genome	Technically feasible, achieves cell-type restricted expression, can create models with stable expression, unrestricted by lncRNA gene architecture	Cheap and easy to design strategy with a predictable outcome,	Direct targeting of RNA molecules without affecting the genome	Allows the interrogation of the biological function of RNA modifications	Stable gene editing and highly specific	Technically easy and cheap to design, several optimizations to CNS delivery
**Limitations**	Restricted by lncRNA gene architecture, off-target effects of gRNA, risk altering expression of adjacent genes	Restricted by lncRNA gene architecture, off-target effects of gRNA, risk altering expression of adjacent genes, transient effect	Does not maintain endogenous mechanisms, cannot replicate cis-acting lncRNA function, correct localization and post-transcriptional modifications should be confirmed	Limited to NGG PAM-sequences, possible off-target effects.	gRNA design depends on the secondary structure of the target RNA and the presence of PFS sequence	gRNA design depends on the secondary structure of the target RNA and the presence of PFS sequence	Experimentally expensive and time-consuming	High risks of off-target effects, toxicity, assay-dependent efficiency, limited in targeting nuclear transcripts

*N/A* not available or does not apply.

In loss-of-function studies, the design of targeting strategies is often impeded by the deficiencies in lncRNA annotation. This poses special difficulties in the case of lncRNAs that overlap with other transcripts, such as intronic lncRNAs with insufficiently defined 5′ and 3′ ends, where it is difficult to predict the impact of lncRNA disruption on the host gene splicing processing. Inaccuracy in transcript annotation can also be a major drawback for full functional recovery by ectopic expression of the lncRNA. In addition, ribosome footprinting, ribo-seq and proteomic analysis have made it increasingly clear that RNAs formerly annotated as noncoding transcripts may indeed be translated, even though noncoding functions could still be present and related to the transcript levels. An accurate distinction between the coding and the noncoding contribution to the ascribed function may be complex but necessary for the full understanding of a particular RNA molecule in the brain. In the specific case of circRNAs, which are highly abundant in the CNS, their particular biogenesis, susceptibility to targeting, coding potential, and requirements for optimal exogenous production are features that differ greatly from linear lncRNAs and need to be analyzed carefully (specific guidelines in circular RNA research by experts in the field have been recently published [160]). Fortunately, a plethora of bioinformatic tools are being developed to predict lncRNA structure, binding partners, and function. This is fundamental for their proper classification and annotation, and to help identify disease-relevant noncoding species, as well as to assist in the experimental design for functional testing. Of special relevance is the development of machine learning-based approaches with increasing accuracy in the prediction of disease-associated lncRNAs, which envisages a future where the automated interpretation of diagnoses will routinely include considerations about lncRNA species [161].

When considering clinical applications, disruption or enhancement of lncRNA function in the brain shares some difficulties with the use of coding genes as therapeutic targets. Namely, the BBB is a burden for successful in vivo delivery by relatively non-invasive methods (such as systemic distribution through intravenous injection). In addition, small RNA oligonucleotides generated to target disease-associated lncRNAs can be rapidly degraded by endogenous nucleases in circulation, and their modified or DNA-based alternatives are still unable to efficiently reach different brain regions [162]. However, the development of tailored nanocarriers designed to increase bioavailability and delivery across the BBB is an intense area of study, and the optimization of such vehicles will greatly impact the feasibility of both coding and noncoding targeting approaches [163]. These approaches certainly predict an exciting boost for clinically-relevant research on disease-causing lncRNAs in the brain.

Currently, ongoing clinical trials primarily focus on assessing lncRNAs as biomarker molecules, with limited exploration of their use as tools or direct targets for therapy. These trials are predominantly conducted in the context of cancer, where lncRNAs are being investigated as potential indicators of disease stage and progression. Additionally, a few clinical trials are evaluating the utility of lncRNAs as biomarkers in neurological conditions. For instance, in acute ischemic stroke, two clinical trials (NCT04175691 and NCT04230785) are measuring circulating circular RNAs (circRNAs), microRNAs (miRNAs), and lncRNAs to assess the prognostic value of these RNA molecules in untreated compared to endovascularly treatment stroke patients, shedding light on the ability of RNAs to predict stroke outcomes. In a separate clinical trial (NCT03152630), next-generation sequencing technologies are being employed to investigate circulating lncRNA expression and exosomal RNAs in individuals with and without cognitive dysfunction or dementia. The objective is to use these RNA profiles as predictive markers for neurodegenerative conditions in patients who have previously experienced a stroke.

While the current clinical landscape primarily focuses on lncRNAs as biomarkers, it is worth noting that preclinical research is increasingly exploring the manipulation of lncRNAs for therapeutic purposes. As our understanding of the functional roles of lncRNAs continues to expand and preclinical approaches advance, it is likely that we will see a transition towards the translation of lncRNA-based therapeutics into clinical trials. This shift holds promise for the development of innovative treatment strategies that target lncRNAs in various diseases, potentially transforming the field of precision medicine.

## Data Availability

All data produced for this manuscript is incorporated within the published article.
